# ^18^F-FAPI-04 Outperforms ^18^F-FDG PET/CT in Clinical Assessments of Patients with Pancreatic Adenocarcinoma

**DOI:** 10.2967/jnumed.123.266283

**Published:** 2024-02

**Authors:** Xiang Li, Na Lu, Lili Lin, Yiwen Chen, Shuye Yang, Huatao Wang, Xinyuan Liu, Chengyi Wu, Xing Xue, Xinhui Su, Xueli Bai, Tingbo Liang

**Affiliations:** 1 Department of Hepatobiliary and Pancreatic Surgery, First Affiliated Hospital, School of Medicine, Zhejiang University, Hangzhou, China;; 2 Zhejiang Provincial Key Laboratory of Pancreatic Disease, Hangzhou, China;; 3 Department of Nuclear Medicine, First Affiliated Hospital, School of Medicine, Zhejiang University, Hangzhou, China;; 4 Department of Radiology, First Affiliated Hospital, School of Medicine, Zhejiang University, Hangzhou, China; and; 5 Zhejiang University Cancer Center, Hangzhou, China

**Keywords:** ^18^F-FAPI-04, ^18^F-FDG, PET/CT, pancreatic ductal adenocarcinoma, staging

## Abstract

Accurate diagnosis and staging are crucial for selecting treatment for patients with pancreatic ductal adenocarcinoma (PDAC). The desmoplastic responses associated with PDAC are often characterized by hypometabolism. Here, we investigated ^18^F-fibroblast activation protein inhibitor (FAPI)–04 PET/CT in evaluation of PDAC and compared the findings with those obtained using ^18^F-FDG. **Methods:** Sixty-two PDAC patients underwent ^18^F-FAPI-04 PET/CT and ^18^F-FDG PET/CT. Identification of primary lesions, lymph node (LN) metastasis, and distant metastasis (DM) by these methods was evaluated, and TNM staging was performed. Correlation between SUV_max_ of the primary lesion and treatment response was explored in patients who received systemic therapy. **Results:** ^18^F-FAPI-04 PET/CT identified all patients with PDAC; ^18^F-FDG PET/CT missed 1 patient. Tracer uptake was higher in ^18^F-FAPI-04 PET/CT than in ^18^F-FDG PET/CT in primary tumors (10.63 vs. 2.87, *P* < 0.0001), LN metastasis (2.90 vs. 1.43, *P* < 0.0001), and DM (liver, 6.11 vs. 3.10, *P* = 0.002; peritoneal, 4.70 vs. 2.08, *P* = 0.015). The methods showed no significant difference in the T staging category, but the N and M values were significantly higher for ^18^F-FAPI-04 PET/CT than for ^18^F-FDG PET/CT (*P* = 0.002 and 0.008, respectively). Thus, 14 patients were upgraded, and only 1 patient was downgraded, by ^18^F-FAPI-04 PET/CT compared with ^18^F-FDG PET/CT. A high SUV_max_ of the primary tumor did not correlate with treatment response for either ^18^F-FAPI-04 or ^18^F-FDG. **Conclusion:** ^18^F-FAPI-04 PET/CT performed better than ^18^F-FDG PET/CT in identification of primary tumors, LN metastasis, and DM and in TNM staging of PDAC.

## Visual Abstract

**Figure:**
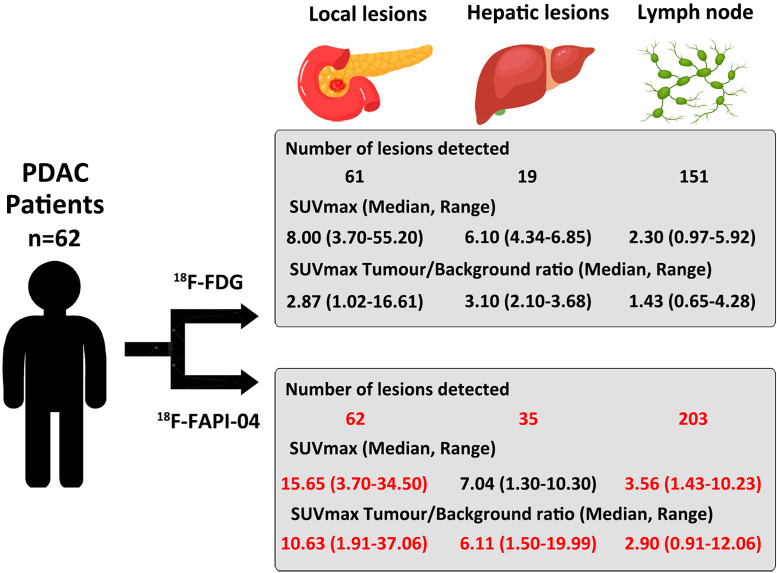

Pancreatic ductal adenocarcinoma (PDAC) is one of the most lethal malignancies (1). Accurate diagnosis and initial staging are crucial for optimal treatment selection. Imaging techniques, including CT and MRI, are the most frequently used methods for tumor detection, staging, treatment response evaluation, and tumor surveillance (2*,*^3^). CT scans, which offer good resolution and wide anatomic coverage, are routinely used for tumor staging and assessment of resectability. Both local and distant diseases can be assessed in a single session (4). However, the detection of micrometastases with CT scans remains a major challenge. MRI has proved to be outstanding for detection of small lesions, including identification of local pancreatic tumors and screening for hepatic or peritoneal micrometastases. However, screening-range limitations restrict the application of MRI in the detection of distant metastases (DMs) (5).

PET/CT is a hybrid imaging technique with wide anatomic coverage that allows the depiction of all possible small metastases throughout the body. ^18^F-FDG is the most widely used radiotracer for PET/CT. Although hypermetabolic tumors are known to demonstrate particularly high ^18^F-FDG uptake, the desmoplastic reaction associated with PDAC usually shows hypometabolic characteristics, which is a well-known limitation of ^18^F-FDG PET/CT in PDAC diagnosis and staging (6–8).

The tumor cells in PDACs exist within a dense stroma, which is composed of an extracellular matrix, vasculature, and cancer-associated fibroblasts (9). Fibroblast activation protein (FAP) is a membrane protease that is highly expressed on the surface of cancer-associated fibroblasts (10*,*^11^). Therefore, a radioactively labeled FAP inhibitor (FAPI) is a promising PET tracer in PDAC (12*,*^13^). Moreover, PDAC is expected to show intensive uptake of ^68^Ga-conjugated FAPI (^68^Ga-FAPI). The clinical value of ^68^Ga-FAPI for PDAC has been preliminarily investigated, and the studies have shown promising results (14*,*^15^).

Nevertheless, storage and long-distance transit of ^68^Ga are difficult because of its relatively short half-life. In addition, the availability of ^68^Ga-labeled tracers from ^68^Ge/^68^Ga generators is limited. In contrast, ^18^F is the most widely used radionuclide in PET; therefore, it can be easily produced in larger doses and delivered over longer distances at a relatively lower cost than ^68^Ga. Thus, ^18^F-labeled FAPI-targeting tracers are strongly desired in clinical practice (16). However, the advantages of ^18^F-AlF-NOTA-FAPI-04 (^18^F-FAPI-04) over ^18^F-FDG have not yet been systematically evaluated in PDAC. Our purpose was to explore the potential efficacy of ^18^F-FAPI-04 PET/CT for PDAC tumor staging and compare the results with those obtained using ^18^F-FDG PET/CT.

## MATERIALS AND METHODS

### Enrollment and Treatment

Sixty-two patients with PDAC were enrolled prospectively between August 2021 and February 2023 at the First Affiliated Hospital, School of Medicine, Zhejiang University. The hospital’s ethics committee approved this study (NCT05884463; ClinicalTrials.gov), and all patients gave written informed consent. For comparative analyses, both ^18^F-FAPI-04 PET/CT and ^18^F-FDG PET/CT were performed at enrollment. The inclusion criteria were as follows: patients who were suspected to have PDAC by radiologic imaging; patients who had scheduled paired ^18^F-FAPI-04 PET/CT and ^18^F-FDG PET/CT for metastasis screening, recurrence confirmation, or tumor staging; and patients who were willing to participate in clinical trials and who signed an informed-consent form. The exclusion criteria were as follows: patients who were not pathologically diagnosed as PDAC, pregnant patients, and patients with the inability or unwillingness of the research participant, parent, or legal representative to provide written informed consent. After systemic treatment, surgical treatment was performed if the patients met the criteria for a conversion operation. The decision to complete preoperative PET/CT was based on the patient’s willingness. The treatment response was evaluated bimonthly according to RECIST version 1.1. Final clinical staging was conducted by our tumor board and based on clinical, pathologic, and all imaging data.

### Radiopharmaceuticals

^18^F-FAPI-04 was prepared as described previously (17*,*^18^). The NOTA-FAPI-04 precursor was purchased from Beijing PET Technology Co. Ltd. ^18^F was produced from a medical cyclotron (Siemens Medical Solutions). The synthesis of ^18^F-FAPI-04 was performed in an AllInOne synthesis module (Trasis). The final product was reconstituted in saline and passed through a 0.22-μm syringe filter (Pall Corp.). The radiochemical purity of ^18^F-FAPI-04 was analyzed by radio–high-performance liquid chromatography (1200 series; Agilent) and was more than 95%. ^18^F-FDG was synthesized automatically and routinely in a ^18^F-FDG synthesizer module (FDG4 Explora; Siemens) and was purified to radiochemical purity of more than 95% before clinical use.

### PET/CT Imaging

PET/CT imaging with both ^18^F-FAPI-04 and ^18^F-FDG was performed on a PET/CT scanner (Biograph version 600; Siemens Healthineers). All images were acquired from top of skull to mid thigh 60–90 min after intravenous administration of ^18^F-FAPI-04 or ^18^F-FDG at a dose of 3.7–4.44 MBq/kg (0.1–0.12 mCi/kg). Fasting and normal blood glucose levels were obtained for ^18^F-FDG PET/CT. ^18^F-FAPI-04 PET/CT and ^18^F-FDG PET/CT were performed within 2 wk, and both were conducted before treatment. The PET scan was performed with 3 min/frame three-dimensional acquisition. The CT parameters were 120 kV, 160 mA, pitch of 1.3, slice thickness of 2.5 mm, and rotation time of 0.5 s, and these were used to conduct PET attenuation correction. PET images were reconstructed using a Siemens workstation (syngo.via Client 4.1) with TrueX plus time of flight (UltraHD PET [Siemens]; 10 iterations, 5 subsets, gaussian filter with full width at half maximum of 4 mm, 440 × 440 matrix).

### PET/CT Image Analysis

Two nuclear medicine physicians, both of whom have more than 10 y of experience in nuclear oncology, independently analyzed all images using a MedExsystem nuclear medical information system (MedEx Technology Limited Corp.), and discordant results were resolved by consensus. Image interpretation included visual analysis and quantitative assessments. Focal ^18^F-FAPI-04 or ^18^F-FDG accumulations showing activity higher than the background, except for physiologic uptake, were considered potential positive lesions. The uptake of ^18^F-FAPI-04 or ^18^F-FDG in primary tumors and metastatic lesions was semiquantified by SUV_max_. To ensure that SUV_max_ was relatively comparable, the tumor-to-background (T/B) ratio was performed according to the following formula: T/B ratio = tumor SUV_max_/background SUV_mean_. Average SUV_mean_ of the liver was set as the background to SUV_max_ of the local tumor. Background SUV_mean_ of hepatic or bone metastasis was average SUV_mean_ of normal liver tissue or bone tissue, respectively. For lymph node (LN), pleural, and peritoneal lesions, background SUV_mean_ was set as average SUV_mean_ of the descending aorta. Average background SUV_mean_ was calculated for 3 random regions. If there were fewer than 5 lesions in a single organ, all lesions were quantitatively assessed. Otherwise, the 5 lesions with the highest activity were quantitatively evaluated.

### Statistical Analysis

Continuous variables were expressed as mean ± SD, whereas categoric variables were expressed as frequency and proportion. ^18^F-FAPI-04 and ^18^F-FDG uptake were compared using the paired *t* test. The McNemar–Bowker test was used to assess significant differences between ^18^F-FAPI-04 and ^18^F-FDG PET/CT for TNM staging. All statistical analyses were conducted using SPSS (version 18.0; IBM).

## RESULTS

### Participant Characteristics

All patients were pathologically diagnosed as showing PDAC by biopsy or surgery. Fifty-eight patients were newly diagnosed and treatment-naïve, whereas the other 4 patients underwent PET/CT for restaging after initial treatment. Our cohort consisted of 43 men and 19 women, with a median age of 63 y. Finally, 54 patients received further treatment at our institution, including surgery treatment (*n* = 4) and systemic treatment (*n* = 50). In addition, 48 patients who received systemic treatment underwent radiologic response evaluation; these patients were included to investigate the value of the 2 tracers in treatment response prediction. More details about the patients’ concurrent symptoms, comorbidities, tumor location, carbohydrate antigen 19-9 values, and other pertinent data are recorded in Supplemental Table 1 (supplemental materials are available at http://jnm.snmjournals.org).

### Adverse Events

^18^F-FAPI-04 and ^18^F-FDG were tolerated by all participants without physical discomfort or adverse effects.

### Diagnostic Performance of ^18^F-FAPI-04 and ^18^F-FDG in Primary Tumors

**FIGURE 1.**
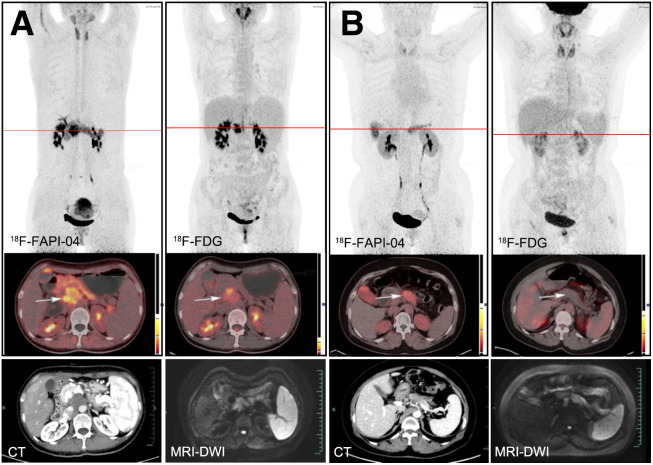
Typical PET (top), PET/CT (middle), and CT and MR (bottom) images of primary tumor obtained using 2 tracers in representative patients (A and B). Tumor is marked by arrows. DWI = diffusion-weighted imaging.

^18^F-FDG PET/CT showed a sensitivity of 98.4% (61/62 patients) for identification of primary tumors, whereas ^18^F-FAPI-04 PET/CT identified all local lesions (62/62 patients). ^18^F-FAPI-04 SUV_max_ was almost 2 times greater than ^18^F-FDG SUV_max_, increasing from a mean of 8.00 (range, 3.70–55.20) to 15.65 (range, 3.70–34.50) in the semiquantitative parametric analysis (Table 1) and showing that the uptake of ^18^F-FAPI-04 in primary tumors was significantly greater than that of ^18^F-FDG (*P* < 0.0001). The difference of T/B ratio in uptake between ^18^F-FAPI-04 and ^18^F-FDG was more pronounced (10.63 vs. 2.87, *P* < 0.0001). The typical PET/CT images obtained with the 2 tracers and the corresponding CT/MR images are shown in Figure 1.

**TABLE 1.** Comparison of ^18^F-FDG and ^18^F-FAPI Uptake in Lesions

Parameter	Patients (*n*)	^18^F-FDG uptake				^18^F-FAPI-04 uptake				^18^F-FDG SUV vs. ^18^F-FAPI-04 SUV *P* value
		Median SUV_max_	Range of SUV_max_	Patients (*n*)	Positive lesions (*n*)	Median SUV_max_	Range of SUV_max_	Patients (*n*)	Positive lesions (*n*)	
Primary tumor	62			61	61			62	62	
Original		8.00	3.70–55.20			15.65	3.70–34.50			<0.0001
T/B ratio		2.87	1.02–16.61			10.63	1.91–37.06			<0.0001
LNs	44			40	151			44	203	
Original		2.30	0.97–5.92			3.56	1.43–10.23			<0.0001
T/B ratio		1.43	0.65–4.28			2.90	0.91–12.06			<0.0001
Metastasis										
Liver	12			5	19			12	35	
Original		6.10	4.34–6.85			7.04	1.30–10.30			0.388
T/B ratio		3.10	2.10–3.68			6.11	1.50–19.99			0.002
Peritoneal	12			11	103			12	158	
Original		2.82	2.10–7.94			6.00	3.10–10.83			0.016
T/B ratio		2.08	0.99–4.14			4.70	1.90–12.85			0.015
Bone	3			1	4			3	6	
Original		8.92	ND			7.00	4.90–13.25			0.925
T/B ratio		7.28	ND			8.00	5.76–40.60			0.678
Pleural	1			1	18			1	26	
Original		3.00	ND			6.00	ND			ND
T/B ratio		4.31	ND			7.32	ND			ND

ND = not determined.

### Diagnostic Performance of ^18^F-FAPI-04 and ^18^F-FDG for LN Assessments

**FIGURE 2.**
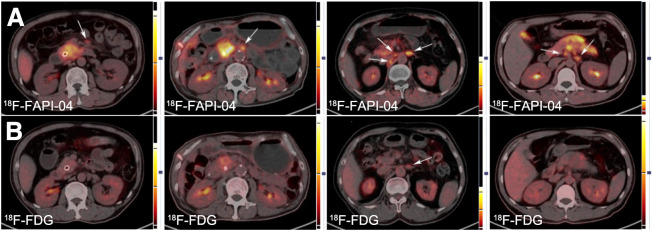
Typical LN PET/CT images obtained with ^18^F-FAPI-04 (A) and ^18^F-FDG (B) from 4 patients. Lesion is marked by arrows.

In total, 44 patients showed large LN shadowing with high metabolism after performing PET/CT. Among these, 40 patients showed abnormal LN findings on ^18^F-FDG PET/CT, whereas the remaining 4 patients showed suggestive findings on ^18^F-FAPI-04 PET/CT alone (Table 1). ^18^F-FAPI-04 showed an obvious advantage over ^18^F-FDG in terms of the number of positive LNs identified (203 vs. 151). In the semiquantitative study, median SUV_max_ and maximum SUV_max_ for ^18^F-FAPI-04 uptake were 3.56 and 10.32, respectively, which were higher than the values for ^18^F-FDG (median SUV_max_, 2.30; maximum SUV_max_, 5.92), with a *P* value of less than 0.0001. The difference in uptake between ^18^F-FAPI-04 and ^18^F-FDG was more pronounced in the T/B ratio (2.90 vs. 1.43, *P* < 0.0001). The 2 examination approaches showed a substantial difference for the identification of LN metastases (Fig. 2).

### Diagnostic Performance of ^18^F-FAPI-04 and ^18^F-FDG for DM

**FIGURE 3.**
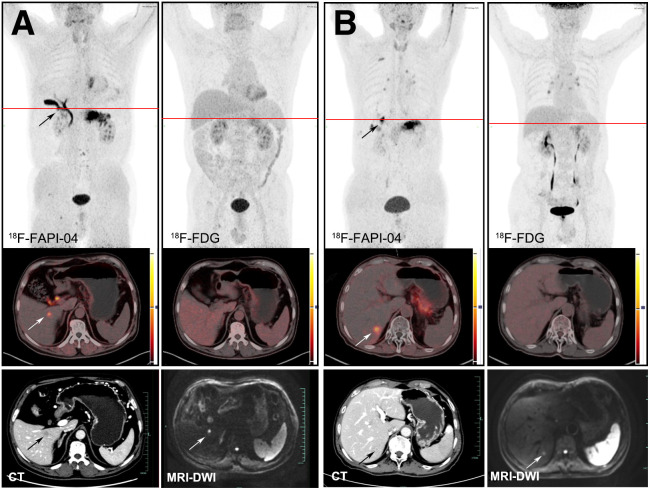
Typical PET (top), PET/CT (middle), and CT and MR (bottom) images of hepatic metastases obtained using 2 tracers in 2 patients (A and B). Lesion is marked by arrows. DWI = diffusion-weighted imaging.

**FIGURE 4.**
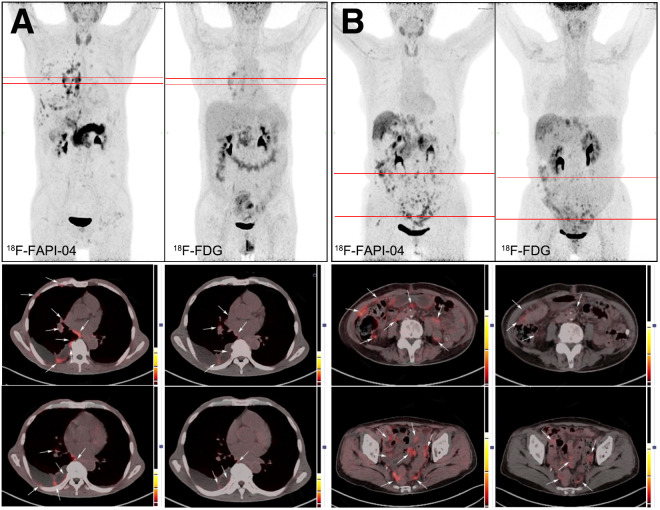
Typical PET (top) and PET/CT (middle and bottom) images showing pleural (A) and peritoneal (B) metastasis obtained with 2 tracers. Axial PET/CT images correspond to red lines in coronal PET images. Lesion is marked by arrows.

The data for the number of positive hepatic, peritoneal, bone, and pleural metastases and the semiquantitative parameters of ^18^F-FAPI-04 PET/CT and ^18^F-FDG PET/CT are presented in Table 1. ^18^F-FDG and ^18^F-FAPI-04 confirmed hepatic metastasis in 5 and 12 patients, respectively, implying that ^18^F-FAPI-04 surpassed ^18^F-FDG in the detection of hepatic lesions. SUV_max_ in hepatic metastases was slightly higher for ^18^F-FAPI-04 than for ^18^F-FDG (7.04 vs. 6.10), but the difference was not significant (*P* = 0.388). To exclude background effects, the T/B ratio of ^18^F-FAPI-04 was higher than that of ^18^F-FDG (6.11 vs. 3.10, *P* = 0.002). Altogether, ^18^F-FAPI-04 PET/CT showed better sensitivity and accuracy than ^18^F-FDG PET/CT for detection of hepatic metastases. The images of representative cases are presented in Figure 3. Similar results were obtained for patients with peritoneal metastasis. Although the sample size of patients with bone or pleural lesions was limited, ^18^F-FAPI-04 PET/CT demonstrated higher detection rates of these lesions than did ^18^F-FDG PET/CT (Fig. 4).

### TNM Staging

Sixty-two patients were staged according to the eighth edition American Joint Committee on Cancer tumor staging criteria (Supplemental Table 2). The distribution of T staging was similar between the 2 tracers. Assessment of vascular involvement based on enhanced CT was more accurate than that based on PET/CT. Therefore, the T4 staging proportion based on CT/MRI (58.1%) was significantly greater than that based on PET/CT.

N staging was more variable between ^18^F-FDG and ^18^F-FAPI-04. Four patients without LN metastases, according to ^18^F-FDG, were categorized as N1 by ^18^F-FAPI-04, and 11 patients who were categorized as N1 according to ^18^F-FDG were categorized as N2 by ^18^F-FAPI-04. Moreover, preoperative ^18^F-FAPI-04 PET/CT was performed in 13 patients. Pathologic examination confirmed 290 LNs. Of these, 23 positive LNs were confirmed in 6 patients. LN involvement included 18 true-positive, 26 false-positive, 241 true-negative, and 5 false-negative findings with ^18^F-FAPI-04 PET/CT. The sensitivity, specificity, and accuracy for the diagnosis of LN metastasis were 78.3%, 90.3%, and 89.3%, respectively (Supplemental Table 3).

^18^F-FDG PET/CT revealed DM in 17 patients, whereas ^18^F-FAPI-04 PET/CT showed DM in 24 patients. ^18^F-FAPI-04 PET/CT upgraded the M stage in 7 patients. Five of them were confirmed to have hepatic metastasis by ^18^F-FAPI-04 PET/CT, whereas the remaining 2 patients were found to have peritoneal metastases and bone metastases.

**FIGURE 5.**
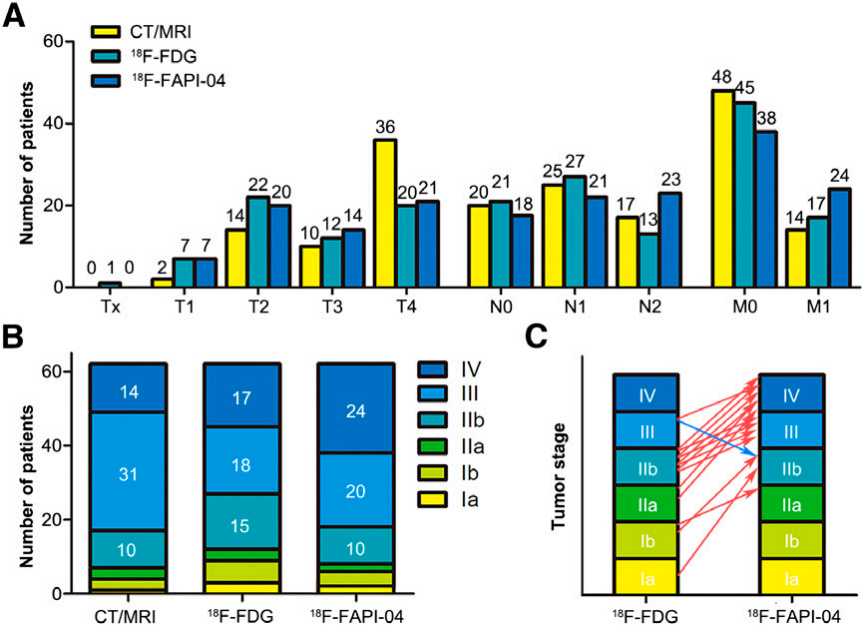
Staging based on CT/MRI, ^18^F-FAPI-04 PET/CT, and ^18^F-FDG PET. Shown are number of patients in T, N, and M categories (A); prognostic stage groups based on CT/MRI, ^18^F-FAPI-04 PET/CT, and ^18^F-FDG PET/CT (B); and differences in prognostic staging of patients between 2 tracers (C).

Figure 5 illustrates how, in comparison with ^18^F-FDG PET/CT, ^18^F-FAPI-04 PET/CT upgraded the staging of 14 patients: 1 from Ia to IIb, 1 from Ib to IIa, 1 from Ib to IIb, 2 from IIa to IV, 4 from IIb to III, 4 from IIb to IV, and 1 from III to IV. However, only 1 patient was downstaged from III to IIb after ^18^F-FAPI-04 PET/CT (Supplemental Tables 4 and 5).

### Treatment Response Evaluation

**FIGURE 6.**
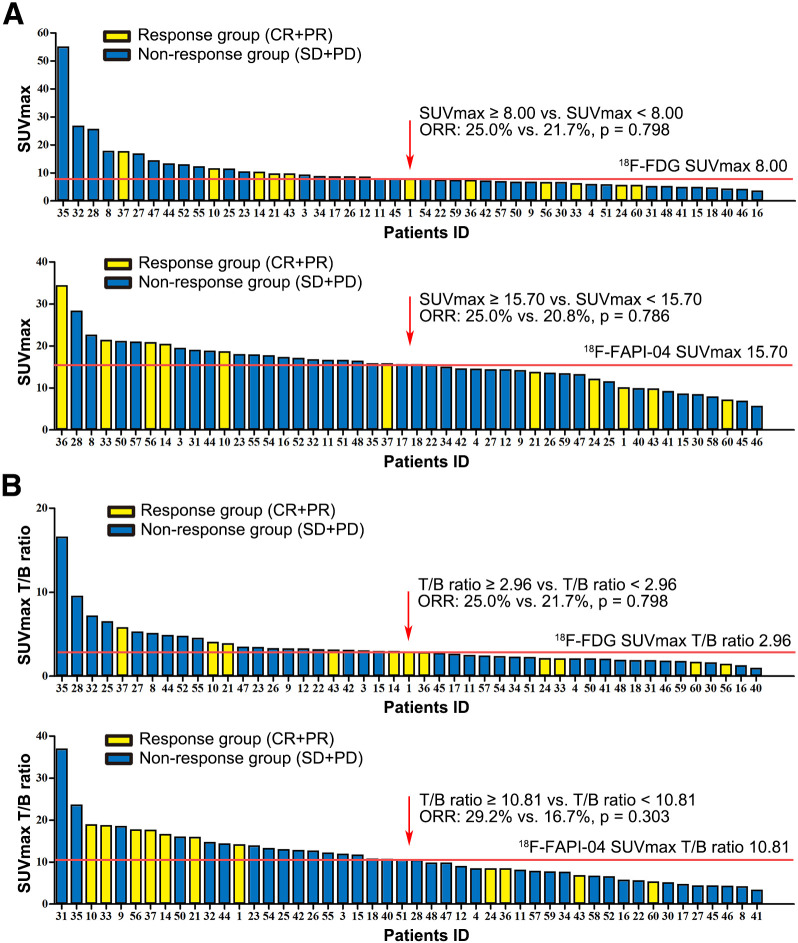
Relationship between SUV_max_ and treatment response. Shown are ^18^F-FDG and ^18^F-FAPI-04 SUV_max_ (A) and ^18^F-FDG SUV_max_ and ^18^F-FAPI-04 SUV_max_ T/B ratio (B) based on primary tumor and related treatment response in patients. CR+PR = complete response and partial response; ORR = objective response rate; SD+PD = stable disease and progressive disease.

Forty-eight patients received systemic treatment, and the best treatment response was recorded. The correlations between SUV_max_ or T/B ratio and response were analyzed (Fig. 6). Median SUV_max_ and median T/B ratio values of ^18^F-FDG and ^18^F-FAPI-04, respectively, were identified as the cutoff values. Patients were divided into response group (complete and partial response) and nonresponse group (stable and progressive disease). Patients showing higher uptake of ^18^F-FDG (≥8.00) or ^18^F-FAPI (≥15.70) showed response rates similar to those of patients with lower SUV_max_ (^18^F-FDG, 25.0% vs. 21.7%, *P* = 0.798; ^18^F-FAPI, 25.0% vs. 20.8%, *P* = 0.786). Similarly to SUV_max_, a lower ^18^F-FAPI-04 T/B ratio was not significantly associated with an increased response rate (29.2% vs. 16.7%, *P* = 0.303). Therefore, the level of uptake of ^18^F-FAPI-04 or ^18^F-FDG failed to predict the response to systemic treatment.

## DISCUSSION

Diagnosis and proper staging based on imaging assessments are essential for choosing treatment plans for tumor patients. Unfortunately, CT, MRI, and other routinely used imaging examinations frequently fall short in various aspects, especially in assessments of PDAC. Our results demonstrate that ^18^F-FAPI-04 PET/CT is significantly superior to ^18^F-FDG PET/CT in detecting both primary and metastatic lesions.

The most widely used PET tracer is ^18^F-FDG, which relies on functional activity to distinguish metabolically active proliferative lesions, because tumors frequently accumulate ^18^F-FDG (19). However, the use of ^18^F-FDG PET/CT for the detection and staging of suspected PDAC remains debatable (6). The sensitivity of ^18^F-FDG PET/CT in the initial diagnosis of PDAC ranges from 73% to 94% (20), and our study results were slightly higher than this range (∼98.4%). In contrast to ^18^F-FDG, the tracer ^18^F-FAPI-04 offers a new method for identification of malignancies (11*,*^12^). Pang et al. (12) reported that ^68^Ga-FAPI was more sensitive than ^18^F-FDG for the identification of PDAC, although their study included only 26 patients. Our study had a larger sample size: 62 PDAC patients were enrolled. In our investigation, ^18^F-FAPI-04 had a remarkably higher T/B ratio than that of ^18^F-FDG, although its identification of primary tumors was similar to that of ^18^F-FDG. A previous study demonstrated that ^68^Ga-FAPI PET/CT can be used to determine the expression of FAP and further guide ^177^Lu-FAPI radionuclide therapy in patients with breast cancer (21). Our study confirmed that PDAC shows high uptake of ^18^F-FAPI-04, which may also indirectly represent the high expression of FAP in PDAC, giving a diagnostic and clinical strategy for treatment.

LN metastasis is one of the independent factors affecting the prognosis (22). Particular importance should be placed on preoperative examination and prediction of LN status. However, ^18^F-FDG shows limited utility in assessing LN metastasis. In a study by Wang et al. (23), the accuracy of ^18^F-FDG in determining LN metastasis of PDAC in 160 patients was only 39.4%. The authors theorized that this may be related to LN size. Positive LNs often have a large number of cancer-associated fibroblasts, which can be combined with ^18^F-FAPI for visualization (24). In our investigation, ^18^F-FAPI-04 showed an obvious advantage over ^18^F-FDG in terms of the number of positive LNs detected and higher tracer uptake, suggesting that ^18^F-FAPI-04 is more sensitive than ^18^F-FDG in the identification of metastatic LNs. In our study, 13 patients who received tumor resection underwent ^18^F-FAPI-04 PET/CT preoperatively, and 290 LNs were confirmed with pathologic examination. The sensitivity, specificity, and accuracy of the diagnosis of LN metastasis based on ^18^F-FAPI-04 PET/CT were 78.3%, 90.3%, and 89.3%, respectively, which implies that ^18^F-FAPI-04 PET/CT performed well in detecting metastatic LNs. However, we did not find pathologic evidence to support the advantages of ^18^F-FAPI-04 PET/CT over ^18^F-FDG PET/CT in the assessment of LN metastasis, because preoperative paired PET/CT was not essential according to our study design.

The ^18^F-FDG detection findings for hepatic metastases are equally unsatisfactory (25*,*^26^). Pang et al. (12) and Deng et al. (15) have demonstrated that ^68^Ga-FAPI is more effective than ^18^F-FDG in distinguishing hepatic metastases from PDAC and gastrointestinal cancers, respectively. Similarly, hepatic metastasis was indicated by ^18^F-FDG alone in only 5 patients in our study. ^18^F-FAPI-04 and ^18^F-FDG had a similar SUV_max_. High uptake of ^18^F-FDG in the liver background may cover the uptake in some micrometastases. In contrast, ^18^F-FAPI-04 showed better background contrast with lower uptake in the liver. Similar results were observed for peritoneal, bone, and pleural lesions. Thus, ^18^F-FAPI-04 upstaged 14 patients in comparison with ^18^F-FDG findings. Although detection of metastatic lesions by PET/CT has improved greatly, the assessment of vascular involvement based on enhanced CT is more accurate.

Some studies have already shown that the high expression of FAP on cancer-associated fibroblasts is strongly associated with aggressive tumor behavior and poor prognoses (27*,*^28^). PDAC patients with moderate or strong FAP expression experience shorter overall survival than those with negative or weak expression (29). Pancreatic tumor cells are known to exist within a dense stroma, which accounts for nearly 90% of the tumor mass. Therefore, ^18^F-FAPI-04 uptake is better than ^18^F-FDG uptake as a possible indicator of tumor prognosis. Moreover, the presence of an abundant stromal compartment may create a physical barrier to decrease microvascularity and drug delivery in the tumor, thereby reducing the sensitivity to systemic therapy. In this regard, the visualization of FAP expression using ^18^F-FAPI-04 seems to be a promising approach to predict the response to systemic treatment. In our study, we evaluated the correlation between ^18^F-FAPI-04 uptake and treatment response, but no significant difference was observed in the objective response rate in relation to differences in ^18^F-FAPI-04 versus ^18^F-FDG uptake. This may result from the limitation of the radiologic response for PDAC: it is difficult to observe obvious tumor shrinkage even in cases showing significant tumor cell regression. Because all stages of PDAC were included in our study and some patients underwent conversion surgery after treatment, we failed to analyze the correlation of SUV_max_ with progression survival, which is an obvious limitation. Thus, additional studies are required to validate the prognostic value of ^18^F-FAPI-04.

This study had some other limitations. First, we included only patients with pathologically diagnosed PDAC, and the assessment of ^18^F-FAPI-04 was limited to evaluating the sensitivity of this technique, with no assessments of the specificity and other indicators. Disease lesions such as those presenting in IgG4-related disease are known to show significant fibrosis, as well as the potential for high ^18^F-FAPI uptake (30). Furthermore, pathologic evidence to support the advantages of ^18^F-FAPI-04 over ^18^F-FDG in the assessment of LN metastasis was insufficient, because all enrolled patients underwent ^18^F-FAPI-04 and ^18^F-FDG PET/CT at diagnosis, and preoperative PET/CT was not essential according to our study design.

## CONCLUSION

Our results show that ^18^F-FAPI-04 performed better than ^18^F-FDG in identifying the primary tumor, LN metastasis, and DM and for TNM staging in PDAC. In the future, ^18^F-FAPI-04 PET/CT may play a greater role in the actual clinical management of PDAC.

## DISCLOSURE

This work was supported by the National Key Research and Development Program (2019YFC1316000 to Tingbo Liang), the National Natural Science Foundation of China (U20A20378, 81830089, and 82188102 to Tingbo Liang; 81871925 and 82071867 to Xueli Bai; 82071965 to Xinhui Su; 82071916 to Xiang Li; and 82172859 to Yiwen Chen), the Key Research and Development Program of Zhejiang Province (2019C03019 to Tingbo Liang and 2020C03117 to Xueli Bai), the Fundamental Research Funds for the Zhejiang Provincial Universities (2021XZZX031 to Xueli Bai), and Huadong Medicine Joint Funds of the Zhejiang Provincial Natural Science Foundation of China (LHDMZ22H300010 to Xinhui Su). No other potential conflict of interest relevant to this article was reported.

## Notes

KEY POINTS **QUESTION:** Is ^18^F-FAPI-04 PET/CT more effective than ^18^F-FDG PET/CT at identifying primary lesions, LN metastases, and DMs of PDAC? **PERTINENT FINDINGS:** In this 62-patient prospective study, ^18^F-FAPI-04 PET/CT showed performance superior to that of ^18^F-FDG PET/CT in the detection of primary lesions and metastases of PDAC and eventually upgraded the TNM stage in 14 patients. **IMPLICATIONS FOR PATIENT CARE:** ^18^F-FAPI-04 PET/CT is expected to assist in the detection of PDAC, offer more accurate staging, and help patients choose surgery or other treatment options.
